# Perspective: One and one make three - leveraging the synergy of imaging and biomechanics in osteoarthritis research and care

**DOI:** 10.1016/j.ostima.2026.100469

**Published:** 2026-07-11

**Authors:** Wouter Schallig, Niels B.J. Dur, Mariska G.H. Wesseling, Rianne A. van der Heijden, Jaap Harlaar, Edwin H.G. Oei, Erin M. Macri

**Affiliations:** a Department of Radiology & Nuclear Medicine, Erasmus MC University Medical Center Rotterdam, Postbus 2040, 3000 CA Rotterdam, Netherlands; b Department of Orthopaedics and Sports Medicine Rotterdam, Erasmus MC University Medical Center, Postbus 2040, 3000 CA Rotterdam, Netherlands

**Keywords:** Pathomechanics, Joint loading, Multimodal assessment, Computational modeling, Functional imaging, Joint health, Osteoarthritis, Biomechanics

## Abstract

Mechanical stress on joint tissues is a central driver of osteoarthritis (OA), yet our understanding of OA pathomechanics remains incomplete, obstructing etiology-driven personalized treatments. Current joint assessment approaches are largely monodisciplinary. In this perspective we aim to advocate for the integration of imaging and biomechanics to assess joint function in OA research, by exploring their relative strengths and limitations and their potential synergy, towards advancing our understanding of pathomechanics in OA. Traditional structural imaging is often static and non-weightbearing, limiting its ability to elucidate pathomechanical pathways of OA. Meanwhile, biomechanical measurement approaches like motion capture are able to measure human movement during dynamic, weightbearing, and functional activities like walking or squatting, but their accuracy and precision is limited by soft tissue artefacts and modeling assumptions and constraints. We argue for the deliberate integration of imaging and biomechanics modalities to overcome their respective limitations towards a comprehensive understanding of the role of pathomechanics in OA. This can done, for example, via videoradiography-based motion analysis and personalized computational models that use additional imaging outputs from radiographs, CT or MRI. Achieving this vision of integrated multimodal analysis requires coordinated, transdisciplinary collaboration and shared resources to move beyond single-modality paradigms toward an integrated framework for pathomechanical joint health assessment, which subsequently can be used to better understand the complex relationships between mechanics, structure and biology within OA. Studies adopting this integrated approach are limited, yet emerging examples showcase the potential for advancing the field of OA research and, eventually, personalized care.

## Introduction

Mechanical stress on joint tissues has been recognized since at least the 1970s as a major, if not the most important, cause of osteoarthritis (OA) development[[1], [2], [3]]. Nevertheless, we as researchers often need to be reminded of this[[1], [2], [3]]. Our understanding of OA pathomechanics remains incomplete, and this may in part explain the ongoing shortage of effective treatments. Core recommended treatments, namely exercise therapy, education, and weight management, only yield small between-group improvements in pain and function, and are not universally effective[4]. This limited success may, in part, be due to a one-size-fits-all approach to a heterogeneous disease. Phenotyping according to distinct pathophysiological or pain mechanisms (e.g., mechanical, metabolic, inflammatory) has been proposed as a strategy to identify subsets of patients who may benefit from more targeted interventions[5]. Meaningful phenotyping requires a coordinated approach, taking into account the most important drivers of OA, to overcome current methodological limitations in OA research.

OA is a complex disease involving intertwined biological, structural, and mechanical components[6]. The complexity of OA necessitates a holistic, systems biology approach to understand how multiple factors and pathways interact to cause loss of homeostasis and ultimately whole organ failure. Yet OA etiology is generally studied within monodisciplinary silos such as imaging[7], or biomechanics[8]. Imaging modalities provide critical insight into joint and tissue structure and pathophysiology, while biomechanical assessments quantify joint alignment, movement patterns, and loading during functional tasks. The convergence of imaging and biomechanics within OA research remains limited, but a quickly evolving technical landscape has the field of OA poised for an important shift in how we use these modalities to understand OA. Therefore, in this perspective our aim was to advocate for the integration of imaging and biomechanics to assess joint function in OA research, by exploring their relative strengths and limitations and their potential synergy. To advance our understanding of the role of pathomechanics in OA, we posit that this multimodal approach is critical to further elucidate OA etiology, identify and characterize OA phenotypes, and ultimately achieve effective personalized care.

## Imaging joint health assessment: capabilities and limitations

Imaging is a critical tool to assess the development and progression of OA. The main advantage of imaging is that underlying bone or soft tissues can be directly visualized (Table 1), providing detailed insights into structural and biological features that may contribute to, and in turn be influenced by, altered biomechanics. Serial imaging through time enables objective monitoring of disease progression, including evaluating how abnormal loading patterns or mechanical interventions affect cartilage structure and joint health. Imaging modalities (e.g., radiographs, computed tomography (CT), and magnetic resonance imaging (MRI)) also enable us to measure how structural or biological OA features (e.g., bony alignment, morphology) influence or predict movement and joint loading patterns and relate those to OA[9]. The main limitations of imaging in this context are that functional movement and loading patterns are difficult to measure, since traditional imaging often uses planar sequences and smaller fields of view (i.e., relative to large motion capture spaces in biomechanics labs), with joints often in static, supine, or unloaded positions. This makes it difficult to quantify functional joint mechanics or fully interpret imaging in the context of OA, in which relevant pathomechanics and habitual joint loading patterns occur in three dimensions (3D) and during upright, weightbearing, dynamic functional movements[[10], [11], [12]].

**Table 1:** Commonly used imaging modalities for measuring OA features.

	Position*	Weightbearing status	Movement	Dimensionality	Constraints	Feasibility
Radiography					Image detector size (single joint, limb, or whole body (EOS)) Any rigs used (e.g. fixed-flexion frame) Device design limits positioning (e.g. C-arm, standing frame) Radiation	Equipment: conventional is inexpensive and widely available; videoradiography and EOS are relatively expensive and not widely available Skills/qualifications: standard radiology, radiation safety Acquisition time: short
Conventional radiography	Supine Standing	NWB FWB	Static	2D/planar		
Video radiography (fluoroscopy)	Supine	FWB	Dynamic	Planar/ Biplanar		
EOS	Standing	FWB	Static	Biplanar with normative-data-informed-3D-digital reconstruction		
Computed tomography CT	Supine	NWB	Static	3D	Image detector size Device design (e.g., CT bore size) Motion artefact Radiation	Equipment: CT common in hospitals; WBCT more specialized/research focused; PCCT expensive, not yet widely available Skills/qualifications: standard radiology, radiation safety Acquisition time: short
Photon counting PCCT	Supine	NWB	Static			
Weightbearing WBCT	Standing	FWB	Static			
4DCT	Supine	NWB	Quasi-dynamic (slow motion only)			
Magnetic Resonance						Equipment: generally expensive vs. other modalities; some instruments are not widely available (e.g., open bore MRI) Skills/qualifications: standard radiology Acquisition time: longer (3–8 min per scan; up to 90 min total session)
Traditional closed-bore	Supine	NWB PWB with rig	Static Limited dynamic sequences (e.g., cine, kinematic)	Planar image stacking à 3D digital reconstruction 3D isotropic sequence	Bore diameter Coil size/shape (e.g., limits knee flexion) Magnet strength Acquisition sequence to visualize specific tissues, e.g.: - morphologic (T1w, T2w PDw ± fat saturation) - cartilage composition (T1ρ, T2, T2*) - bone imaging (ZTE, UTE)	
Open-bore	Supine Standing Other	NWB PWB with rig FWB	Static Static-sequential Limited dynamic	Planar image stacking à 3D digital reconstruction 3D isotropic sequence	Open bore design (C-shaped bore vs. totally open) Maintain joint at isocenter during motion Generally lower magnetic field strength, resulting in lower image quality	
Nuclear medicine						Equipment: requires specific infrastructure Skills/qualifications: additional qualifications required Acquisition time: long preparation time (for tracer administration); acquisition time depends on instrument (e.g. PET-MRI); patient protocols during and after tracer exposure
PET (NaF)	Supine	NWB	Static	3D	Radiation (radiotracers)	
SPECT (Tc-99 m)	Supine	NWB	Static			

Abbreviations: FWB full weightbearing; PWB partial weightbearing; NWB non-weightbearing; 2D two dimensional; 3D three dimensional; PDw proton density weighted; NaF Sodium Fluoride; Tc-99 m Technetium-99 m; PET positron emission tomography; SPECT single photon emission computed tomography.

⁎ Considerations for optimizing functional/mechanical demands of the joint include: position (standing/upright is most functional); weightbearing status (full weight bearing is most functional); movement (dynamic is most functional); and dimensionality (3D is most functionally relevant).

There are notable and emerging exceptions to the limitations of traditional imaging approaches. Functionally relevant information can be obtained using imaging instruments or approaches that consider biomechanical principles like physiological loading and movement. One approach is to measure acute response to joint loading, for example using quantitative MRI or PET/MRI *before and immediately after* an imposed loading protocol (e.g., squats) or activities like running or cycling[13]. Although inconsistent changes are shown in quantitative cartilage composition (T2, T1rho relaxation times), there is cartilage volume loss post-loading that is both dose-dependent and transient[13]. Specialized instruments [11,14] or loading rigs [15] permit joint imaging to be performed *while* the individual is either upright and fully loaded, or supine and partially loaded. For example, weightbearing CT[11] and upright MRI[14] have demonstrated that bony malalignment, cartilage deformation, and meniscal extrusion are more pronounced during static loading. In vivo joint motion can also be measured using dynamic imaging approaches like dynamic MRI sequences or 4DCT[10,16]. However, these approaches also have trade-offs, often still limited to planar, non-weightbearing, or slow movements.

## Biomechanical joint health assessment: capabilities and limitations

The biomechanical approach generally starts with motion capture (Table 2). Traditional optical motion capture employs synchronized infrared cameras that track skin-mounted markers on a person as they move to calculate the joint kinematics (i.e., motions). In combination with force plates that measure ground reaction forces, joint kinetics (i.e., moments) can be derived. The advantage of a biomechanical joint assessment is that the person can perform any upright, fully weightbearing, functional, and potentially pain-provoking task that is feasible within the measurement environment. The main limitation of motion capture is its limited accuracy due to soft tissue artefacts. During a functional task, the skin-mounted marker moves with respect to the underlying bone it is supposed to track due to soft tissues (i.e., skin, fat, muscles). This can lead to grossly miscalculating the true position of the underlying bones. For example, knee flexion can be underestimated by up to 20°[17].

**Table 2:** Commonly used motion capture modalities for measuring motion biomechanics in OA.

	Position	Weightbearing status	Movement	Dimensionality	Constraints*	Feasibility
Skin-mounted markers tracked by infrared cameras	Any	FWB	Dynamic, speed and type of movement may be limited by design of lab (e.g. fluoroscopy, use of treadmill)	3D	Soft tissue artefacts affecting accuracy Requires a controlled lab environment Number of cameras affects resolution and marker detection	Equipment: generally expensive vs. other modalities Skills/qualifications: use of equipment; accurate marker placement/anatomic palpation; post-processing skills vary depending on model and software used Acquisition time: 1–2 h total (prep participant and equipment; data acquisition depends on task, 10 s – 2 min per task/trial)
Markerless (video-based)	Any	FWB	Dynamic	2D (single camera) 3D (multiple cameras)	Sensitive to environment Lower accuracy and precision (e.g., tracking errors, joint center estimation)	Equipment: one or two video cameras or smartphones; portable so can use outside of a lab Skills/qualifications: learn software Acquisition time: 2–30 min (no marker placement required)
Wearable sensors	Any	FWB	Dynamic	3D	Very sensitive to anatomical calibration and sensor drift Soft tissue artifacts affecting accuracy	Equipment: generally expensive vs. other modalities; portable Skills/qualifications: learn software and hardware (i.e. sensors) Acquisition time: 20–30 min (wearable placement and calibration required)

Abbreviations: FWB full weightbearing; 2D two dimensional; 3D three dimensional.

In terms of scale, far fewer motion capture labs exist in research and clinical settings compared to imaging facilities, so these labs may be more difficult to access. Wearable sensors and markerless (smartphone or video-based) systems have emerged as accessible, lower-cost alternatives to traditional motion capture labs that provide the added advantage of being portable, thus permitting biomechanics to be measured outside of a lab, including in natural environments[7,18]. While the accuracy and precision of these technologies are lower than those of traditional motion capture systems, this is expected to improve as algorithms are refined and adapted to OA populations. For researchers with limited biomechanics or engineering expertise, or limited resources, these lower fidelity systems may serve as feasible entry points into biomechanical data collection.

The most relevant biomechanical outcome in the OA population is joint loading during functional activities. Since this cannot be measured directly in native joints, computational modeling techniques, such as musculoskeletal models and finite-element models, are used to calculate joint and cartilage loading. Musculoskeletal models primarily use motion capture and force plate data applied to a scaled, general model that contains information about musculoskeletal structures (i.e., bones, joints, muscles, ligaments) linked with mathematical equations and a set of assumptions and constraints. For example, most musculoskeletal models are based on average characteristics of small samples of healthy white males (e.g., average dimensions of male pelvises). These generic models are then linearly scaled and assumed to be valid for individuals with OA, who are typically older and predominantly women. While assumptions and constraints are inherent to any model, developing more anatomically detailed or population-specific models requires substantial expertise, time, and computational resources. As a result, researchers often rely on simplified, readily available models, even though this may compromise the accuracy and precision of the estimated joint loading.

## Multimodal joint health assessment: potential and challenges

**Fig. 1:**
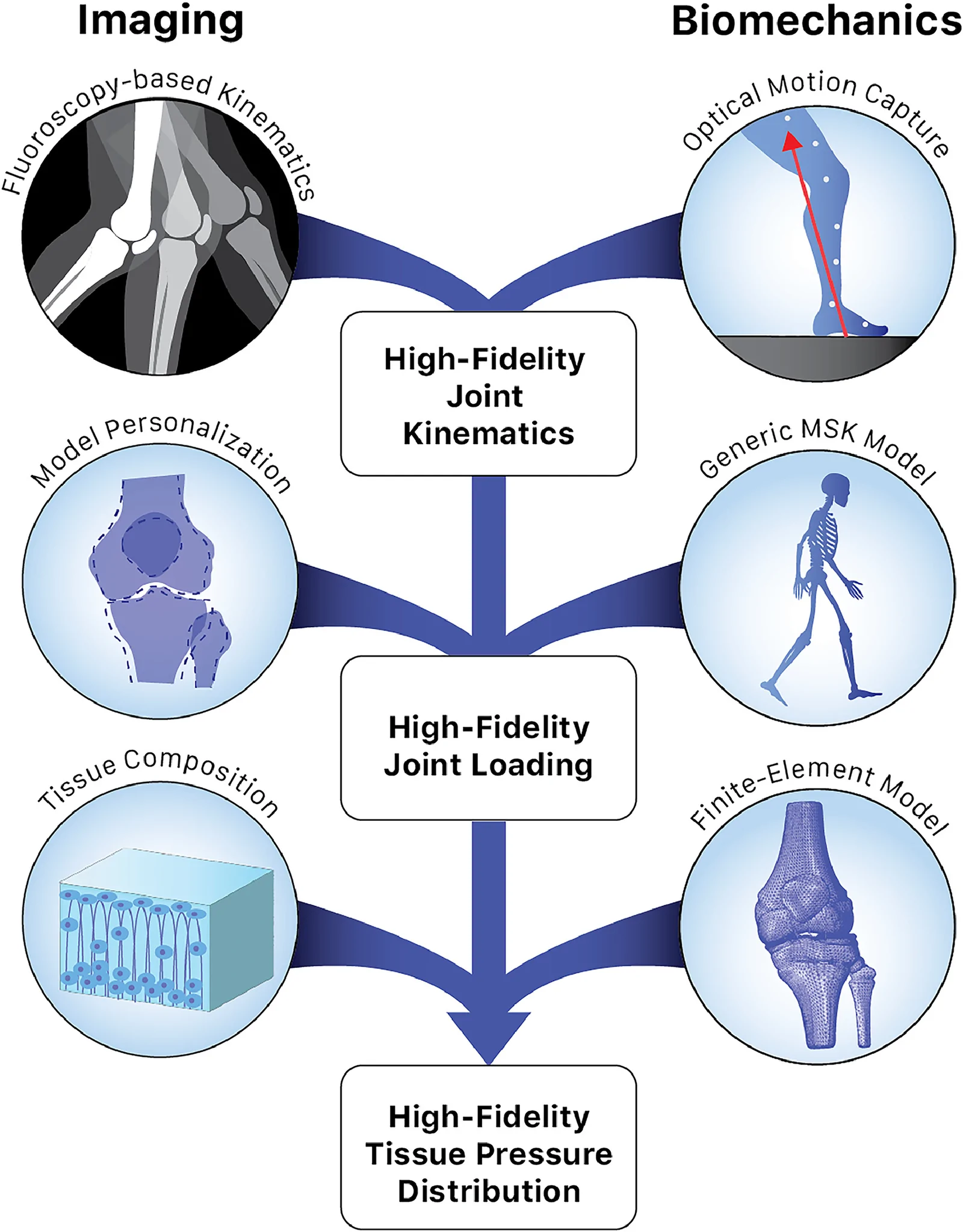
Schematic overview of a pipeline integrating imaging and biomechanics to assess high-fidelity joint pathomechanics. The visual example for model personalization showcases personalized bone geometry, however this could also involve e.g. bone alignment, muscle attachment points, muscle moment arms, muscle architecture, and ligament characteristics. Similarly, for the tissue composition, this could also involve e.g. cartilage segmentations.

The deliberate integration of multiple imaging- and biomechanics-derived inputs into high-fidelity, personalized models represents a promising opportunity to overcome the respective limitations of the single-modality approaches outlined above (Fig. 1). Without imaging, we lack the precision diagnostics, and insight into anatomical/structural causes and the compositional consequences of pathological mechanics. Without biomechanics, we lack the functional context of how people move and load their joints, which can both cause or be a consequence of tissue and joint structure and biology. This integrated approach can yield two major benefits to OA research. First, it enables studying the interplay between joint pathomechanics and joint structure and physiology, thus elucidating the complex mechanisms underlying OA development and progression. Second, combining imaging and biomechanics enables a more accurate, precise, and functional assessment of the pathomechanical component of joint health. Studies fully adopting this transdisciplinary approach are limited, yet they showcase the potential for advancing the field of OA research and care.

Collecting both imaging and biomechanics assessments within a single study, even if not fully integrating these modalities, adds clinical and contextual information that either modality alone lacks. For example, a proof-of-concept study included 16 women with knee OA who underwent asynchronous morphological and compositional MRI before and after walking and cycling in a motion capture lab [19]. Bone shape (imaging) and knee joint reaction forces (biomechanics) both predicted cartilage deformation patterns. Another example is a randomized clinical trial of a one-year personalized gait retraining program (vs. sham) that yielded 7.5% greater reduction in knee adduction moment (a proxy for load), 12 points (out of 100) greater reduction in pain; and −3.7 ms less medial femoral cartilage degeneration on T1rho [20]. Including both imaging and biomechanics in this trial provided evidence that may explain the mechanism underlying the symptom improvements with treatment.

Early efforts to integrate modalities was initially achieved through asynchronous data collection [21]. For example, CT or MRI images can be acquired (separately/asynchronously from motion capture) and images segmented to create subject-specific 3D bone models, from which measures like bone morphology and joint contact areas can be extracted. This information is then integrated with motion capture data by imputing it into a musculoskeletal model. This approach yields more personalized estimates of joint angles, moments, or cartilage stress during gait than can be estimated using generic models typically used for motion capture analysis [21].

Synchronous imaging and biomechanics data collection is now also possible, with a growing number of motion capture laboratories embedding single or biplanar videoradiography (i.e., fluoroscopy) directly into the motion capture setup. Videoradiography directly visualizes bones and thus overcomes the dependency on skin-mounted markers to capture joint motion. Additional imaging is needed to create 3D bone models used to register (i.e., match) the 3D object to the 2D videoradiography images, which yields sub-millimeter accuracy of bone kinematics[12]. These accurate bone kinematics are then integrated into musculoskeletal models to improve estimates of joint loading parameters. While videoradiography does involve ionizing radiation, the dose is relatively low; for example, in our lab up to 180 s of exposure to the knees (15 Hz) involves 0.1 mSv. An example study evaluated cartilage contact characteristics during gait in individuals with and without obesity [22], They synchronously collected motion capture with embedded biplanar fluoroscopy, and asynchronously acquired MRI to derive 3D bone models and cartilage surface models to add to the models. They found medial tibiofemoral contact locations were shifted medially in individuals with obesity, creating altered loading conditions that may contribute to higher OA rates in this population.

Increasingly sophisticated and personalized computational models are achieved by combining general musculoskeletal models with imaging-derived model parameters including bone geometries, bone alignment, muscle attachment points, muscle moment arms, muscle architecture, and ligament characteristics. These imaging inputs could yield iteratively more accurate estimations of clinically relevant loading characteristics like joint contact forces or relative distribution of forces between the medial and lateral tibiofemoral compartments [23]. The output of the musculoskeletal models (e.g., joint reaction forces) can subsequently serve as inputs for participant-specific finite element models that focus on biomechanics at the tissue scale (e.g., cartilage). These models can be personalized by adding information about cartilage material properties as derived from, for example, T_2_ relaxometry MRI, leading to estimating properties like stress distributions, stiffness, or degradation over time [24].

Integrated models will likely lead to identifying and validating new biomarkers or surrogate markers of OA disease itself. This can be accomplished by, for example, comparing cartilage pressure distribution maps (derived from finite element models) with compositional MRI imaging or ^18^F-Sodiumfluoride PET/MRI to map cartilage or bone metabolism, an approach we are currently working towards in our own lab. Potentially supported by artificial intelligence, we can also look for patterns like clustering of mechanical features with other OA features (e.g., structural, biological, demographic) to better understand how different features co-occur during the onset and development of OA. This could help to crystalize our understanding of OA phenotype(s) (e.g., predominantly mechanically or inflammation driven), while also placing it all within the context of a complex, heterogeneous disease. In this perspective, we mainly approach OA from a joint-level perspective, however we should not forget to also collaborate with our fundamental science colleagues to develop a comprehensive mechanobiological model of OA that factors in (sub)cellular OA processes, like mechanotransduction pathways that regulate tissue homeostasis and degeneration [3].

Many research groups are not yet prepared for the shift towards integrated multi-modal joint assessment. Three key barriers limit the development and widespread adoption of such approaches, despite their promising utility. First, transdisciplinary research is required, which means not just working in multidisciplinary environments, but actually developing and applying cross-functional skills while working in teams that bring together clinical, imaging, and engineering expertise and the systems biology perspective, providing a unified framework for understanding OA pathomechanics. Second, the equipment and space requirements are extensive and often state-of-the art, meaning they are not routinely available in research institutions. Third, linking multiple inputs across modalities is both technically and computationally demanding.

Methods are continuously evolving to improve how multi-model data can be linked, registered, calibrated, and synchronized. Simultaneously, pipelines are becoming more streamlined, for example through automating time-consuming steps like image segmentation and registration. We encourage OA researchers to consider how they can move towards collecting both imaging and biomechanics assessments within a single study, even if data are acquired using lower-fidelity instruments or the measures are initially analyzed separately. Including both modalities within a study helps to facilitate the shift towards integrated multimodal modeling. Doing so prepares research teams to adapt their skills and resources to the shifting technology-driven landscape. It also provides data needed to (i) improve our understanding of how these modalities interact, which informs algorithm development; and (ii) serve as training datasets for automating, building and validating integrated models.

## Outlook

The deliberate integration of imaging and biomechanical modalities is a crucial next step in OA research. This emerging approach aims to overcome the respective limitations of the two acting alone. Together they can improve the precision and accuracy of our mechanical models while also ensuring the clinical relevance of dynamic, functional, weightbearing activities that underlie our daily loading patterns. Moreover, this approach yields information to help understand the complex relationships between mechanics, structure and biology within OA development. Understanding this will further elucidate OA etiology, better characterize OA phenotypes, and elucidate the mechanisms underlying symptomatic and structural responses to treatment, ultimately leading to more effective personalized care. Achieving our vision requires a coordinated transdisciplinary approach and shared resources to move beyond single-modality paradigms toward an integrated framework for understanding OA pathomechanics.

## Funding

WS was partly funded by an Erasmus MC2 Young Investigator Grant and NWA-ORC research programme grant (LoaD project, #NWA1389.20.009) from the Dutch Research Council (NWO). NBJD and EMM received salary support from a ZonMW Open grant (MOBI project, #09120011910052). RAvH received salary support from a ZonMw VENI personal grant (#09150162310051).

## Declaration of generative AI in scientific writing

The coauthors declare that no generative AI was used in the writing of this manuscript.

## Declaration of competing interest

The authors declare the following financial interests/personal relationships which may be considered as potential competing interests: Wouter Schallig reports financial support was provided by Dutch Research Council. Niels Dur reports financial support was provided by Netherlands Organisation for Health Research and Development. Erin Macri reports financial support was provided by Netherlands Organisation for Health Research and Development. Rianne van der Heijden reports financial support was provided by Netherlands Organisation for Health Research and Development. If there are other authors, they declare that they have no known competing financial interests or personal relationships that could have appeared to influence the work reported in this paper.
